# An Overview of Essential Microelements and Common Metallic Nanoparticles and Their Effects on Male Fertility

**DOI:** 10.3390/ijerph191711066

**Published:** 2022-09-04

**Authors:** Ryszard Maciejewski, Elżbieta Radzikowska-Büchner, Wojciech Flieger, Kinga Kulczycka, Jacek Baj, Alicja Forma, Jolanta Flieger

**Affiliations:** 1 Department of Anatomy, Medical University of Lublin, 20-090 Lublin, Poland; 2 Department of Plastic, Reconstructive and Maxillary Surgery, CSK MSWiA, 02-507 Warszawa, Poland; 3 Institute of Health Sciences, John Paul II Catholic University of Lublin, 20-708 Lublin, Poland; 4 Department of Forensic Medicine, Medical University of Lublin, ul. Jaczewskiego 8B, 20-090 Lublin, Poland; 5 Department of Analytical Chemistry, Medical University of Lublin, Chodźki 4A, 20-093 Lublin, Poland

**Keywords:** male fertility, reproduction, essential metals, nanoparticles, toxicity, environmental factor, public health

## Abstract

Numerous factors affect reproduction, including stress, diet, obesity, the use of stimulants, or exposure to toxins, along with heavy elements (lead, silver, cadmium, uranium, vanadium, mercury, arsenic). Metals, like other xenotoxins, can cause infertility through, e.g., impairment of endocrine function and gametogenesis or excess production of reactive oxygen species (ROS). The advancement of nanotechnology has created another hazard to human safety through exposure to metals in the form of nanomaterials (NMs). Nanoparticles (NPs) exhibit a specific ability to penetrate cell membranes and biological barriers in the human body. These ultra-fine particles (<100 nm) can enter the human body through the respiratory tract, food, skin, injection, or implantation. Once absorbed, NPs are transported to various organs through the blood or lymph. Absorbed NPs, thanks to ultrahigh reactivity compared to bulk materials in microscale size, disrupt the homeostasis of the body as a result of interaction with biological molecules such as DNA, lipids, and proteins; interfering with the functioning of cells, organs, and physiological systems; and leading to severe pathological dysfunctions. Over the past decades, much research has been performed on the reproductive effects of essential trace elements. The research hypothesis that disturbances in the metabolism of trace elements are one of the many causes of infertility has been unquestionably confirmed. This review examines the complex reproductive risks for men regarding the exposure to potentially harmless xenobiotics based on a series of 298 articles over the past 30 years. The research was conducted using PubMed, Web of Science, and Scopus databases searching for papers devoted to in vivo and in vitro studies related to the influence of essential elements (iron, selenium, manganese, cobalt, zinc, copper, and molybdenum) and widely used metallic NPs on male reproduction potential.

## 1. Introduction

Infertility affects about 15% of couples of childbearing age and has now become a major public health problem. The above fact is linked to numerous factors, e.g., obesity rates; cardiovascular diseases; hormone-dependent tumors; developmental disorders; chronic childhood diseases; early onset of puberty; altered gender ratio and maternal age; infections of the reproductive system; diet; addictions; stress, which is usually a reaction to mental or emotional pressure; and the accumulation of toxins in the body [1,2,3]. Over the past half-century, we have seen a downward trend in men’s reproductive health. Epidemiological studies have shown a systematic deterioration of the quality of male sperm [4]. One of the important reasons for this is toxic environmental factors, exposure to chemicals, heavy metals, and air pollution [5]. Even small doses of environmental toxins have been characterized as “acting similarly as uncontrolled drugs” for humans [6]. The impact of environmental toxins on health depends on the exposure time (critical “windows of susceptibility”, such as ‘in utero’, adolescence, and adulthood), dose, and duration of exposure [5,7]. People are constantly exposed to toxic environmental factors, particularly endocrine-disrupting chemicals (EDCs). EDC substances include pesticides (e.g., dichlorodiphenyltrichloroethane [DDT], chlorpyrifos, atrazine, 2,4-dichlorophenoxyacetic acid [2,4-D]), lead (Pb), cadmium (Cd) (children’s products), phenol, bisphenols (food packages, e.g., cans or plastic bottles), brominated flame retardants (electronics and building materials), phthalates (e.g., personal care products and tubing), triclosan (antibacterial agents), and perfluorochemicals (textiles and clothing).

Apart from the fact that EDCs impede reproduction, more and more evidence indicates their participation in the development of obesity, cardiovascular diseases, type 2 diabetes, some cancers, neurodevelopmental disorders, mental diseases, and abnormalities of the genitourinary system [6]. Toxic substances are primarily responsible for the production of reactive oxidation species (ROS) and the impairment of hormonal functions that affect the quality of sperm [8]. However, the mechanism of action of EDC is complex and multidirectional. EDCs can alter cellular processes by binding to nuclear steroid hormone receptors (e.g., ThR and GPR30 thyroid receptor) and activating genomic and non-genomic pathways, activating ion channels, inducing pro-inflammatory cytokines and chemokines, promoting oxidative stress, and altering cell proliferation and differentiation [7]. A growing body of evidence suggests that epigenetic mechanisms are also involved, including DNA methylation, histone modifications, and micro-RNA expression [7]. These, in turn, affect gene expression, cell and tissue function, and disease risk. Given the changes in methylation/demethylation that normally occur during gametogenesis and embryogenesis [9], the effects of EDC may be significant for generation-to-generation reproductive success.

Heavy metals have been confirmed to present negative effects on male fertility. Particularly dangerous is exposure to Pb, Cd, arsenic (As), bismuth (Bi), chromium (Cr), gold (Au), silver (Ag), mercury (Hg), nickel (Ni), i.e., metals commonly found in the human environment. Many reports describe the negative impact of heavy metals on fertility [10,11,12,13,14,15,16]. Toxic metals cause changes in the morphology, qualitative and quantitative parameters of sperm, as well as biochemical and endocrine disorders. Disruption of hormonal homeostasis is associated with the influence of toxins on the endocrine function of the pituitary and hypothalamus in the brain, and the testes, which are the site of spermatogenesis and androgen production.

Given the high prevalence of infertility worldwide [17,18] and the fact that exposure to toxic trace elements is extremely common, growing attention has been given to the potential effects of exposure to toxic trace elements on human fertility [19,20].

The results of the meta-analysis based on data collected by Sun et al. [21] from the PubMed, Web of Science, and Chinese National Knowledge Infrastructure (CNKI) database until 2016 indicate that low-fertility males have higher Pb, Cd, and lower zinc (Zn) levels in their semen. Similarly, Louis [22] suggested a toxic effect of Cd and Pb on reproduction. A systematic review in PubMED for reports published between 1975 and 2017 on the effects of Hg exposure on human fertility [20] unequivocally found that elevated Hg levels were also associated with infertility. Exposure to Hg induced hormonal disruptions, DNA damage to sperm, abnormal sperm morphology, and motility.

Although the meta-analysis studies support the adverse effect of heavy metals on the semen and sperm quality on fertility, individual studies seem to be no longer conclusive or consistent. For example, most authors describe higher concentrations of Cd and Pb in the sperm plasma [23,24,25], while the study by Mendiola et al. [26] does not confirm the existence of significant differences between infertility and fertility in the concentration of Pb, Cd, and Hg in sperm. Similarly, Garcia-Fortea et al. [27] noticed a positive relationship between the number of mature oocytes and the concentration of Pb in a woman’s hair.

Another example is selenium (Se), the consumption of which has been considered beneficial for human health for many years [28,29]. Even recent case–control studies have suggested a protective effect of Se on human fertility [30,31]. However, being over-zealous in increasing Se intake sometimes has negative consequences, reminding us that Se was originally regarded as a toxic element [32].

To protect sperm from oxidative stress, seminal fluid (SF) has a defense system in the form of enzymatic antioxidants, including superoxide dismutase (SOD), glutathione peroxidase (GPx), and catalase (CAT). Trace metals, such as iron (Fe), Zn, and Se, are also assigned the role of components with antioxidant activity [33]. In general, essential metals, especially Zn, Se, manganese (Mn), Fe, copper (Cu), molybdenum (Mo), and cobalt (Co), are considered beneficial or even protective for male reproductive functions, but in excess, also cause adverse effects [15,34].

The advancement of nanotechnology has created another threat to human safety through exposure to metals in the form of nanomaterials (NMs). Due to their common occurrence in the environment and the fact that they can freely pass through placental barriers and the blood–testicle barrier [35,36], nanoparticles (NPs) pose a serious threat to the reproductive process of humans and animals. Ultrafine particles directly affect reproductive cells, the internal organs, and the endocrine system (i.e., hypothalamus–pituitary–gonad–sperm axis, HPG-S [37]. Nanomaterial (NM) reproductive toxicity refers to the stage of embryo formation [38] and is a consequence of adverse effects on the structure and function of the reproductive organs. Developmental toxicity related to NMs, on the other hand, concerns structural or functional changes that interfere with the differentiation and development of the embryo [39]. Consequently, there is a risk of structural defects, fetal growth retardation, various functional/behavioral abnormalities, and ultimately spontaneous abortion. The exact mechanism of the reproductive toxicity of xenobiotics such as NPs is complex, but one of the key causes appears to be oxidative stress, mitochondrial dysfuncton, and related complications.

Human reproductive health is a complex problem that includes gonadal steroidogenesis, sperm quality, female and male fertility, placental function, the effects of pregnancy, polycystic ovarian syndrome, endometriosis, uterine fibroids, and menopause. Much of the information we have about the health effects of different chemicals comes from animal models, occupational exposure, environmental accidents, and epidemiological data from different populations. Higher doses and short-term exposures are used in animal models, which is not applicable in typical human exposure situations. In addition, human research is associated with epidemiological limitations and stringent requirements for clinical trials.

The current review provides up-to-date knowledge of the effects of essential microelements and metallic NPs on male reproductive toxicity. The review was developed due to the need to constantly monitor and describe the problem from different perspectives and to highlight current research trends. Taking into account the inevitable environmental exposure to hardly biodegradable metals and NPs, which can have catastrophic consequences for the life of the individual and the survival of the species, is necessary for the most precise risk estimation.

**Figure 1:**
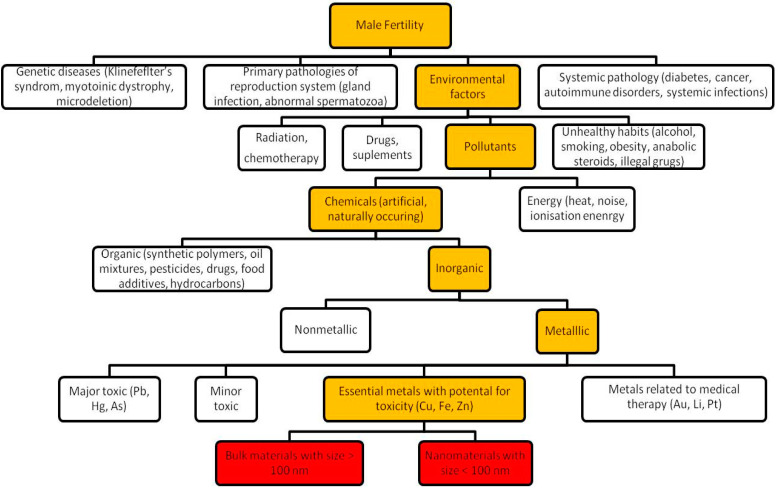
Causes of male infertility. This review is devoted to essential metals with potential toxicity and covers bulk materials and nanomaterials. Orange boxes show the connection of infertility with the subject of this review.

This review focuses on the literature on reproductive nanotoxicity and the possible beneficial or dangerous effects of essential trace metals (Se, Zn, Fe, Co, Cu, Mn, Mo) on male fertility. This study was inspired by individual reported cases of men who had higher levels of microelements such as Zn, Cr, and Cu and lower concentrations of Fe in semen, which were associated with lower sperm motility and serious DNA damage [40]. To our knowledge, no one has previously collected such data in one study. Publications that investigated the relationship between essential metals and NPs on male fertility were extracted from openly available scientific literature using the online version of the Pubmed, Web of Science, and Scopus databases in the range of 1990–2022. The review focuses on experimental studies conducted on animal models and cell cultures (in vivo and in vitro). The connection between men’s infertility with the subject of this review is illustrated in Figure 1.

### 1.1. Sources of Metals

Toxic elements such as Cr, Ni, Cd, Pb, and As are commonly found in the anthropogenic environment. They persist in soil, water, and air [41] because they are not biodegradable. People can be exposed to toxic metals and metalloids through inhalation of dust, ingestion with diet, and direct skin contact [42,43,44]. Cigarette smoke contains about 30 metals, the highest concentrations of which are represented by Cd, As, and Pb. It has been shown that the burden of Cd in smokers is about twice as high as in non-smokers [45]. Alcoholic beverages, including wine, may be contaminated with metals such as aluminum (Al), As, Cd, Cr, Cu, Fe, Mn, Ni, Pb, and Zn in concentrations exceeding the acceptable standards, causing toxic effects, especially in heavy drinkers [46].

We are inevitably exposed to metals due to their ubiquitous nature, wide industrial use, and long-term environmental durability. Information on the sources of exposure to metals has been compiled by the Agency for the Toxic Substances and Diseases Register (ATSDR).

Exposure to metallic contaminants in the form of NPs has increased in recent years. Although humans have come into contact with this type of airborne pollution as a result of sandstorms, volcanic eruptions, and other natural processes, nowadays, due to the advancement of nanotechnology, the risk has increased dramatically [47]. Due to their size, NPs can cross biological membranes and reach many tissues and organs [48,49,50]. It is, therefore, not difficult to notice the serious health risks of NPs. The influence of titanium dioxide (TiO_2_) NPs on the viability and proliferation of mouse Leydig cells; reduction in sperm motility in the presence of Au-NPs; inhibition of postimplantation development of mouse embryos by quantum dots from Cd and Se core; toxicity to the stem of mouse spermatogonia by Ag-NPs, Al-NPs, Mo-NPs; and inhibition of embryo differentiation mouse stem cells by silica NPs were all confirmed [51,52,53,54,55,56,57,58].

It can be observed that numerous published studies describe the effects of exposure to a single metal, although, in reality, we are exposed to both toxic and essential metals that interact with each other. There is little research examining mixed exposures to metals. For example, an increase in the number of spontaneous abortions and low birth weights of children born in a vicinity of a copper smelter in Sweden, which emitted Pb, As compounds, and sulfur dioxide, has been reported [59]. Scientists from Finland reported similar observations regarding the threats from the metal industry [60]. An increased number of miscarriages was noted in women employed in a factory producing Zn and Co compared to the control group recruited from women not related to the metallurgical industry.

Belles et al. [61] examined the effects of co-exposure to the three metallic compounds, lead (II) nitrate (Pb(NO_3_)_2_), methylmercury(II) chloride (CH_3_HgCl), and sodium arsenite (NaAsO_2_), on developmental toxicity in mice as an animal model. Various metal combinations were administered on day 10 of pregnancy. The maternal toxicity in the population exposed to Pb, Hg, and As turned out to be the highest as the metals entered a supra-additive interaction.

A multi-element study on a population of men and women who underwent in vitro fertilization (IVF)/intracytoplasmic semen injection (ICSI) was conducted in China in 2020 [62]. In this research, the relationship between the level of trace elements and the results of IVF was investigated. Serum and follicular fluid (FF) samples and semen plasma were collected from both partners on the day of oocyte collection. The samples were tested for Cr, Ni, As, Se, Cd, and Pb by inductively coupled plasma mass spectrometry (ICP-MS). The authors searched for associations between the levels of toxic and essential trace elements and IVF final results. The study found that the serum Cr level of female partners was inversely correlated with the number of mature oocytes collected (*p* = 0.033). The exposure to toxic elements (Cr, As, and Cd) was related to the results of IVF. In the case of males, exposure to Se may be indirectly associated with better pregnancy outcomes.

Metals can act additively, synergistically, or antagonistically in processes involving absorption, distribution, and excretion (ADME). Toxic metals may compete with essential metals, reducing their concentration in the body or lowering their bioavailability [63,64,65,66]. An example would be the competition between Pb and/or Cd and Zn for the same binding site in enzymes, proteins, and transporters. Such interaction may alter enzyme activity, affect the structure and/or function of cell membranes, induce oxidative stress and apoptosis, and inhibit DNA and RNA synthesis and repair processes in the cell. This can have serious consequences for cell growth, development, and differentiation. On the other hand, essential metals (e.g., Zn, Se) can reduce the absorption and retention of toxic metals and prevent their toxic effects. There are also many other examples of competition between metals. An example is Cd, which can substitute other elements such as Cu, causing a reduction in plasma ceruloplasmin; substituting Zn in metallothionein; and replacement of Fe in ferritin, which causes a decrease in hemoglobin concentration. In turn, lead (Pb) has an affinity for antioxidant enzymes, such as SOD, CAT, and GPx, and competes with their natural cofactors (Zn, Cu, and Se), resulting in enzyme inactivation.

### 1.2. Mechanism of Metal Action

Toxic trace elements can be transported into the cell through cell membranes [67]. The heavy metals function through ROS generation or the inactivation of enzymes usually involved in the antioxidant defense. Currently, it is believed that the main mechanism explaining the loss of fertilization by sperm cells is processes caused by oxidative stress at the cellular level. Oxidative stress (OS)is a state of the overproduction of oxygen and nitrogen free radicals (ROS, RNS) such as HO^•^, ^1^[O]_2_, O_2_^•−^, HOO^•^, ROO^•^, RO^•^, H_2_O_2_, ONOO^−^, NO^•^ [68]. The production of free radicals causes lipid peroxidation (LPO), protein oxidation, and sperm DNA damage [69]. The state of oxidative stress is confirmed by the analysis of biomarkers such as MDA (malondialdehyde); HAE (4-hydroxyalkenals); TBARS (thiobarbituric acid reactive substances assay); protein carbonyl content; 8-hydroxy-2′-deoxyguanosine [8-OhdG]; and the level of enzymes involved in the antioxidant defense system, such as SOD, CAT, GPx, and glutathione reductase (GRd).

The consequences of oxidative stress are cell membrane and DNA damage, leading to cell apoptosis and changes in protein conformation, and the inhibition of enzyme activity. Moreover, toxic elements can disrupt endogenous signaling. It has been confirmed that metals can bind to the estrogen receptors and disrupt hormone signaling, thus causing disturbances in the secretion of sex hormones [70].

### 1.3. Male Fertility Indicators

The quality of sperm largely determines male fertility. The current requirements for semen analysis are described in the guidelines of the World Health Organization (World Health Organization (WHO) 2021) [71]. The spermiogram involves the assessment of various characteristics of the ejaculate fluid. Sperm samples are tested for movement, expressed as percentages, including progressive, non-progressive, and total motility determined under phase contrast microscopy. WHO (2021) reference levels are the following: total motility from 40 to 43%, progressive motility from 29 to 31%, and non-progressive motility at exactly 1%. The reference ranges for immotile sperm and sperm vitality are between 19 and 20% and 50–56%, respectively [71]. Based on the above data, sperm motility can be categorized into fast, progressively motile (a); slow, progressively motile (b); non-progressively motile (c); and immotile (d). Parameters of semen quality involve semen odor, semen volume (1.3–1.5 mL), and total sperm number expressed as 10^6^ per ejaculate (35–40). Furthermore, the morphology of sperm (anomalies of the head, intermediate piece, and tail) and sperm DNA fragmentation (SDF) using different tests (the TUNEL assay, sperm chromatin dispersion assay, Comet assay, and acridine orange flow cytometry assay), oxidative stress (OS), and ROS are evaluated.

The WHO guidelines recommend cytogenetic analysis by the use of fluorescence in situ hybridization (FISH) with the aim of discovering chromosomal aberrations [71].

Parameters of endocrine function in serum are sex hormones, e.g., follicle-stimulating hormone (FSH), luteinizing hormone (LH), testosterone (T), and estradiol. Measurements of trace elements in the seminal fluid may be a better predictor of semen quality than traditional blood measurements in men who are not exposed to work contact [72]. Parameters of seminal plasma include, e.g., metals (Zn, Cd, and others) and fructose.

There are also other tests evaluating, for instance, sperm acrosome reaction, the aniline blue, and chromomycin A3 assays for the evaluation of chromatin condensation or functional analysis of transmembrane ion flux.

It should be underlined that the reference values can not be the only criteria for the diagnosis of normozoospermia, asthenozoospermia, necrozoospermia, and teratozoospermia. To establish the causes of male infertility, we have to consider more parameters [72]. Boitrelle et al. [4], in a comprehensive and critical review, described the Sixth Edition of the WHO manual for human semen analysis (SA).

## 2. Male Reproductive Toxicity of Micronutrient Metals

The seminal plasma contains, in addition to proteins, lipids, and macroelements (sodium (Na), potassium (K), calcium (Ca), magnesium (Mg), phosphorus (P), chlorine (Cl)), several trace elements such as Cu, Zn, and Mn, which are essential for normal spermatogenesis, sperm maturation, and motility, and it is clear that their absence negatively affects the efficiency of fertilization, including capacitation, hyperactivation, and acrosomal response. Metal levels in the seminal fluid are dependent on ion exchange controlled by channels and active transport systems on the cell membrane.

In 2022, Rodríguez-Díaz et al. [73] analyzed the influence of 22 metals in the seminal plasma on sperm quality, as well as fertilization effects, such as the pregnancy rate, implantation, and embryo quality. The importance of Na, K, Ca, and Mg, in particular, has been described [74], in addition to the role of Fe and Zn [75]. Fe and Zn belong to non-enzymatic antioxidants, being enzyme cofactors (enzymatic Fe-catalase), but in excess, they have a negative effect on the quality of sperm and even on the development of the embryo [76]. Similarly, increased levels of Mn and Cu can also be dangerous and deteriorate sperm parameters [77].

There are no reference levels for trace elements in semen or semen plasma, although many studies have looked for a link between the concentration of trace elements in semen or blood of patients and male infertility. The research usually distinguishes the test group, which is compared with the control group recruited from fertile men distinguished by normozoospermia. The concentrations of metals determined in samples taken from healthy men are presented in Table 1. As can be seen, the obtained results were inconsistent.

Disturbances in the levels of metals in the body may have a different background, i.e., incorrect supply, accumulation in the body in case of chronic exposure, or disturbances in metal metabolism. There is a group of genetic disorders associated with defects in proteins/enzymes involved in metal metabolism [91]. The accumulation or deficiency of metals in various tissues can interfere with many biological functions. An example is Wilson’s disease, which is characterized by disturbances in Cu metabolism, and many syndromes that affect the metabolism of Fe, which is one of the key elements for fertility, such as aceruloplasminemia, cerebellar ataxia, hypoceruloplasminemia, Kufor Rakeb syndrome, neurodegeneration with brain accumulation, and HARP syndrome.

This chapter focuses on the literature devoted to the effects of metals on fertility and reproduction in male laboratory animals. This review takes into account studies on the potentially beneficial or dangerous effects represented by essential trace metals (Fe, Co, Cu, Mo, Se, Zn, and Mn).

### 2.1. Zinc

Zn is the second trace mineral in terms of quantity in humans that must be supplied by food. Its beneficial effect on reproduction, especially in men, is known and is described in many review articles [76,77,92,93,94,95].

The importance of Zn for the functions of reproductive organs is emphasized by the fact that its concentration in human seminal plasma is higher compared to other tissues [96]. The amount of Zn in the testes of vertebrates is comparable with that stored in the liver and kidneys [97]. Zn accumulates in the testes during early spermatogenesis. The greatest amount of Zn is found in germ cells. Zn is also detectable in Leydig cells, late-type B spermatogonia, and spermatids. In turn, a lack of Zn is noticed in interstitial tissue and sterol cells. At the cellular level, half of the amount of Zn accumulates in the cytosol, a little less is in the nucleus, and the least amount fills cell membranes [98]. Its function in the nucleus is to stabilize chromatin through S-Zn-S bonds in the protamine structure [99].

The transport of Zn through the physiological membranes takes place via the Zip family and the ZnT family of proteins. Inhibition of Zn transport to the gamete during spermatogenesis causes impairment of testicular function [100,101].

Zn is the cofactor of SOD, an enzyme that is a part of the antioxidant defense system. The antioxidant properties of Zn and the ability to compete with toxic metals are important in protecting the testes against stress factors. Zn is also needed for the proper functioning of the hypothalamus–pituitary–gonadal axis (HPG axis). The synthesis of testosterone in the Leydig cell, and thus the serum testosterone level, depends on Zn [102,103] since Zn is a cofactor of the 5α reductase enzyme transforming testosterone into active 5α dihydrotestosterone [104].

The most important role of Zn in sperm physiology is its participation in spermatogenesis [92,105]. Moreover, Zn has been shown to stimulate sperm capacitation and acrosomal response by activating the epidermal growth factor receptor and the G protein-coupled receptor [106,107]. Zn-induced capacitation signal pathways are also responsible for hyperactive sperm motility [108]. Zn, along with other metals, such as Ca and Mg, ensures normozoospermia, which in turn ensures better-quality embryos. Zn improves the fertilization rate by taking part in the penetration of the sperm into the oocyte to form a mature zygote, as well as in the post-fertilization period. Its beneficial effect on reproduction is confirmed by the fact that as zinc sulfate, it is added to the media used in the production of embryos in vitro [80,109,110,111].

Since it is essential for male fertility, it can be considered a marker in the diagnosis of male infertility [92]. The importance of Zn in maintaining reproductive function, along with the effects of deficiency, is summarized in Table 2. The content of the table was prepared on the basis of data contained in published articles [76,77,80,92,93,94,95,96,97,98,99,100,101,102,103,104,105,106,107,108,109,110,111].

Zn affects both the quality and function of sperm; therefore, its absence reduces the chances of fertilization [80,111]. Zn deficiency is associated with an increase in apoptosis and thus a decrease in sperm count and motility [112].

Zn deficiency has been shown to cause oxidative stress (OS), an inflammatory response, and increased pro-apoptotic signaling (Bax, caspase-3) in germ cells, while anti-apoptotic signals are reduced (Bcl-2) [113]. Zn deficiency is associated with a decreased expression of Zip6 and Zip10 and an altered structure of the seminal tubules with abnormal germ epithelium, regardless of the systemic Zn and testosterone levels [100]. Zn deficiency of Leydig cells may also indirectly contribute to spermatogenesis disorders [114]. Further studies have also shown that Zn deficiency enhances the activation of pro-apoptotic and pro-inflammatory pathways induced by CCl_4_ treatment in testicular cells [115]. The modulation of apoptosis, inflammation, and oxidative stress (OS) can be attributed to the protective effect of Zn against diabetes-induced testicular damage [116].

In a study by Rodríguez-Díaz et al. [73], a significant relationship was discovered between higher Zn levels and better seminal quality. The higher Zn concentrations were observed in men with normal spermiograms with higher sperm counts (95.42 ± 62.39 mg/kg), while pathological changes and decreased concentrations were observed when the Zn concentration was lower (71.18 ± 35.082 mg/kg). The difference reached statistical significance (*p* = 0.044). Other authors obtained similar results [117,118]. Macroscopically, Zn deficiency results in a reduction in the volume and mass of the testicles, contraction of the seminiferous tubules, hypogonadism, and inadequate development of secondary sexual characteristics in humans [118]. Thus, Zn may play a positive role in the process of assisted reproductive treatment [76]. Zn also improved DNA methylation, chromatin integrity, testicular structure, and an increased number of spermatogonia stem cells in a model of testicular toxicity induced by bleomycin etoposide and *cis*-platinum treatment [119].

In 2016, Zhao et al. [95] conducted a systematic analysis of literature data on the subject in PubMed, EMBASE, Science Direct/Elsevier, CNKI, and Cochrane Library. A meta-analysis of 1320 unduplicated studies showed that Zn concentrations in semen in infertile men were significantly lower than in healthy men, and Zn supplementation significantly increased semen volume, sperm motility, and the percentage of normal sperm morphology [95], which was also described in other reports [120,121,122].

Many dietary supplements are used to improve male fertility; however, their effectiveness is debatable. Nevertheless, the effectiveness of Zn supplementation on the increase of semen volume, sperm motility, and morphology was confirmed in a study [123]. Moreover, Zn, in combination with D-aspartic acid and coenzyme Q10, decreased lipid peroxidation in semen [124]. A meta-analysis from 1966 to 2016 of Medline, Scopus, Google Scholar, and Persian databases (SID, Iran medex, Magiran, Medlib, Iran doc) showed that folate plus Zn supplementation has a positive effect on sperm characteristic parameters in subfertile men [125]. The assessment of the composition and effectiveness of supplements used in the Italian market was presented in a report by Garolla et al. [126]. Zn was found to be the most common ingredient in most supplements, along with Se, arginine, coenzyme Q, and folic acid [126].

However, not all studies support a link between Zn in semen plasma and male fertility. Adverse effects of excess Zn on male fertility have rarely been reported. Few reports suggest, though, that an excess of Zn may have a negative effect on sperm quality [92,127]. The relationship between excess Zn in the diet and prostate cancer has also been confirmed in other studies [128]. Studies on 733 incidents and 1228 control cases showed that higher dietary Zn intake might increase the risk of low-grade and localized tumors [128]. Men with a higher genetic susceptibility may also have a higher risk of prostate cancer when taking this nutrient. In a study by Turgut et al. in 2003 [129], the team investigated the effects of excessive Zn consumption on testes and sperm quality in mice. After 3 weeks of supplementation, a statistically significant negative correlation was found between the dose of Zn and the number and motility of sperm. Zn administered in a dose of 2.5 g/100 mL caused decreased sperm motility, degeneration of the seminiferous tubules, and fibrosis in the interstitial tissue. Zn, in the form of complexes, acts as apoptosis inducers in testicular germ cells of *Capra hircus* [130] due to its activation of caspase-3, disruption of mitochondrial membrane potential, DNA fragmentation, and other degenerative changes.

### 2.2. Cobalt

Co is an essential element for mammals that is not accumulated in the body. Data from the literature show that chronic exposure of experimental animals to Co exerts negative effects on the body, including male reproductive organs and fertility. The first such research was completed in 1988 [131], where chronic and acute exposure of male mice to cobalt chloride was studied.

Acute exposure showed no significant changes in reproductive potential. The 7-week observation showed there were no significant changes in epididymal sperm concentration or testicular weight. After a slight decrease, sperm mobility returned to normal one week post-administration.

In contrast, time- and dose-dependent reductions in the above parameters were observed after chronic exposure. Moreover, elevated serum testosterone levels were noted, while FSH and LH levels remained normal. Pedigo et al. [131] hypothesized that Co disrupts the local mechanisms regulating testosterone synthesis and spermatogenesis.

Exposure to exposure during pregnancy and lactation resulted in a reduction in male offspring of reproductive organs, i.e., testicular and epididymal weight, which demonstrates the possibility of Co transfer across the placental barrier and into breast milk [132].

Although there is little research into the effects of Co on male reproductive health, it can be considered a potential risk factor.

### 2.3. Copper

Cu is a trace element that is essential to human health. However, high concentrations of Cu cause serious health problems. It has been reported that massive exposure to Cu may lead to a flu-like condition known as metal fever [133].

In 1956, research began on the influence of Cu on the male reproductive system. The results of the studies were inconclusive, as was the importance of Cu in fertility. However, it has been hypothesized that Cu is involved in sperm motility and may also act on pituitary receptors that control LH release. Cu^2+^ can act as a competitive inhibitor of LH, FSH, and testosterone receptors, inhibiting spermatogenesis [134].

A low level of Cu in seminal fluid occurs in azoospermia and an increase in oligo- and asthenozoospermia. In a study conducted on 95 men recruited from polluted areas, [135] there was a negative correlation of Cu values in seminal plasma with the normal sperm morphology rate. An investigation designed by Mohammed et al. [136] concerned the effect of copper sulfate on the fertility of male white albino rats. Rats were treated orally via stomach tube with 1/10 and 1/5 LD_50_ of CuSO_4_. Results showed a decrease in testes and epididymis weight and a significant increase in sperm abnormalities. Long-term, high-dose Cu^2+^ exposure was found to be negatively correlated with sperm motility, viability and vitality, acrosome intactness, and hyperactivation in a study performed on the vervet monkey (*Chlorocebus aethiops*), the chacma baboon (*Papio ursinus*), and the rhesus monkey (*Macaca mulatta*) used as models for reproductive studies [137].

However, some researchers do not confirm the existence of a negative correlation between the seminal copper level and the number or motility of gametes [138]. These discrepancies may be due to the fact that the Cu concentration in the ejaculate varies over time and is even different in distinct fractions of the same ejaculate. Cu abolishes sperm motility by inhibiting oxidative processes and glucose consumption. This property has been used for contraception in the form of an intrauterine device (IUD) or implantation at various sites in the male system, such as the lumen of the deferens, epididymis, seminal vesicle, and scrotum [139,140].

### 2.4. Manganese

Mn is an essential component of enzymes involved in redox processes, i.e., SOD, pseudo-CAT [141]. Mn presents antioxidant properties, hence the ability to scavenge free peroxide radicals [142] and inhibit LPO [143]. The studies of Cheem et al. [144] in 2009 confirmed the usefulness of (Mn^2+^) for protecting bovine sperm during cryopreservation against lipid peroxidation (LPO) caused by ROS attack. The addition of Mn to the semen showed a protective effect and improved the quality of the semen, i.e., the percentage of motility, the percentage of hypoosmotic edema (HOS), and a decrease in the production of malondialdehyde (MDA) and protein leakage, with the addition of 150 µM and 200 µM Mn^2+^ to the frozen sperm. This fact can be used to improve sperm quality/fertility in in vitro fertilization IVF and artificial insemination.

Mn deficiency is dangerous for health. Some studies have suggested that Mn deficiency causes reproductive impairment [145]. The use of Mn to inhibit damage caused by oxidative stress (OS) is described in a report by Tajaddini et al. [146]. Their studies were carried out on adult mice treated with formaldehyde. Manganese chloride injected intraperitoneally with 5 mg kg^−1^ improved testicular structure and sperm parameters. It should be emphasized, however, that the beneficial effect of Mn on fertility is at low concentrations, while at higher concentrations, it is toxic.

Chronic excessive exposure to Mn causes intoxication, leading to cirrhosis, dystonia, polycythemia, and hypermagnesemia [147]. Mn poisoning can give rise to a clinical picture called manganism, which is associated primarily with syndromes of neurological origin similar to idiopathic Parkinson’s disease [148]. Mn, as a cofactor of mevalonate kinase and farnesyl pyrophosphate synthase, is involved in the synthesis of cholesterol, which is a precursor of sex hormones, and in this sense, can regulate fertility [149].

The reproductive toxicity of Mn has been mainly studied in animals [150]. Environmental exposure to Mn has been shown to be related to the levels of male reproductive hormones. Mn has the ability to upstream genes regulating hypothalamic gonadotropin-releasing hormone (GnRH), which could stimulate prepubertal GnRH release from the hypothalamus [151,152] and the secretion of prepubertal GnRH/LH secretion, accelerating puberty.

Regarding semen quality, it was found that high serum Mn levels were associated with an increased risk of poor sperm quality [153]. Tests were performed on 200 patients who attended an infertility clinic. It was confirmed that high Mn levels were associated with an increased risk of low sperm motility (odds ratio = 5.4; 95% confidence interval = 1.6–17.6) and low sperm concentration (2.4; 1.2–4, 9). The effect of serum Mn levels on fertility was also confirmed by another 2012 study. In the study, semen samples were collected from over 1000 Chinese men. The median serum Mn concentration was 8.2 μg L^−1^. Serum Mn levels were shown to have deleterious effects on sperm morphology and motility [154]. Serum Mn levels greater than 19.40 µg L^−1^ had the most negative effect [154,155]. Mn can induce sperm apoptosis, which is confirmed by the percentage of Annexin V +/PI− spermatozoa as the concentration of Mn increases. Impotence and decreased libido are symptoms of the reproductive system [156]. Another study was conducted on a group of 84 male workers occupationally exposed to Mn compared to a control group (92) [157]. GnRH and LH levels were found to be higher and testosterone levels were lower in the Mn-exposed group. Sperm motility decreased in the Mn-exposed group.

Studies on rabbits have shown that even a single dose of 160 mg MnO_2_/kg administered by inhalation causes degenerative changes in the testes and infertility [158]. For chronic exposure, the dose and duration of exposure are important. While chronic exposure of rats to a dose of about 1000 ppm had no effect on male fertility, a three-fold higher dose caused changes in the form of decreased testicular weight, sperm count, and serum hormone levels (FSH, testosterone) [159]. A 2003 study reported a significant decrease in sperm count and motility in mice orally exposed to manganese acetate for 43 days at a dose of 15.0 and 30.0 mg/kg/day [160].

### 2.5. Selenium

Se is an essential trace element, taking part in many physiological processes as an antioxidant and a component of metabolic changes, e.g., the metabolism of thyroid hormones [161]. Se is recognized as an element that protects against the effects of cadmium (Cd) poisoning. Selenium (Se), as for male reproductive function, can act negatively or be beneficial.

The potential benefits are mainly due to the fact that Se is a cofactor of the antioxidant enzyme (GPx) and a component of two selenoproteins (phospholipid hydroperoxide glutathione peroxidase (PHGPx/GPx4) and selenoprotein P), which are involved in spermatogenesis. Selenium (Se) is a component of selenoproteins, including GPx1, GPx3, mGPx4, cGPx4, and GPx5, which protect against oxidative damage to sperm during the maturation process, while selenoproteins, such as mGPx4 and snGPx4, act as structural components of mature sperm. Se deficiency leads to serious abnormalities in testicular development, spermatogenesis, and sperm quality in the form of loss of motility and morphological abnormalities [162].

The Se-dependent GPx enzyme enables the protection of cell membranes and organelles against peroxidative damage. Se, in combination with vitamin E, has been shown to be effective in improving sperm parameters and pregnancy rates in approximately 700 infertile men with idiopathic asthenoteratospermia [163]. Supplementation with Se (200 μg) in combination with vitamin E (400 units) improved sperm motility, morphology, and induction of spontaneous pregnancy in over 50% of cases after 14 weeks of combined therapy.

This was also confirmed by another study in which a group of 12 infertile men were administered Se in 50 µg (1 capsule) once a day for 3 months [164]. After the therapy, there was a significant increase in sperm count, mobility, viability, normal sperm morphology, and ejaculate volume. The levels of serum Mg, serum FSH, serum LH, serum testosterone, and serum glutathione were significantly increased, and serum MDA was significantly decreased.

This study was repeated a few years later on a group of 50 asthenoteratozoospermic men. Semen samples were incubated with selenium 2 µg mL^−1^ at 37 °C for 2, 4, and 6 h. Parameters such as mobility, viability, and mitochondrial membrane potential improved, and the levels of MDA and DNA fragmentation were significantly lower. Therefore, it was confirmed that Se is effective in protecting the collected sperm (in vitro) against ROS [165].

On the other hand, Se is known to exert toxic effects on various tissues and organs, especially the testes. Studies on rats have shown that chronic exposure to Se is caused by sperm degeneration; intertubular edema; oligospermia; inhibition in activities of testicular steroidogenic enzymes; and a reduction in tubular diameters, tubular areas, and tubular perimeters [166].

The role of Se in male fertility has been described in excellent review articles [167,168,169,170]. It should be emphasized that health benefits relate to a narrow concentration range, which confirms the relationship between biological effects and concentration [171]. Selenosis from overexposure can be acute as well as chronic. The toxic effect of Se is related to the substitution of sulfur in sulfur amino acids and the formation of selenomethionine and selenocysteine, which inactivate succinate dehydrogenase and δ-aminolevulinic acid dehydratase. It has been shown that, in addition to disturbing other physiological functions, it results in decreased fertility [172]. The toxic effect of Se, especially the Se^2−^ and SeH^−^ forms, has also been associated with the mechanism of oxidative stress [173] or the inhibition of selenium methylation by the deactivation of methionine adenosyltransferase [174]. Overexposure to Se induces endoplasmic reticulum (ER) stress through the phosphorylation of JNK and eIF2 proteins [175].

It is also believed that excess Se oxidizes the sulfhydryl groups of proteins and enzymes in mitochondria, blocking energy metabolism in cells [176]. Furthermore, Se influences the Ca^2+^ signaling pathway. Consequences of this disruption could be serious—for instance, DNA breakage in the host cell chromatin, cytoplasmic protein cross-linking, or cytoskeleton destruction.

### 2.6. Iron

Fe is a non-enzymatic antioxidant. It is a CAT cofactor. Elevated levels of this enzyme act as a pro-oxidant and damage sperm due to the formation of hydroxyl radicals and increased lipid peroxidation in plasma membranes [33,177]. Fe detected at a concentration of 5 ppm in seminal fluid decreases sperm motility. Elevated Fe levels were detected in sperm homogenate and seminal plasma of asthenozoospermic and azoospermic men [178]. Elevated Fe levels (0.61 mg/kg) were detected in seminal fluid in pathological spermiograms. However, low iron levels below 0.33 mg/kg also show signs of sperm pathology [73].

### 2.7. Molibden

A 2002 study showed that the oral administration of sodium molybdate to rats at a dose of 10, 30, and 50 mg/kg body weight for 60 days resulted in the accumulation of molybdenum in the testes, epididymides, and seminal vesicles. Exposure to molybdenum (Mo) resulted in a decrease in the weight of reproductive organs and the quality and function of sperm, histopathological changes in the testes, and changes in the level of enzymes such as sorbitol dehydrogenase, LDH, and gamma-glutamyl transpeptidase [179].

## 3. Male Reproductive Toxicity Due to Metallic Nanoparticles

The use of nanotechnology in various fields of industry, medicine, and a variety of consumer applications has caused the widespread use of products made of NMs. The attractiveness of these materials results from their unique properties such as plasma resonance effect, ultra-small size, large surface area to mass ratio, catalytic activity, absorption abilities, and high reactivity [180]. In addition, bringing the matter to the nano scale (1–100 nm) introduces additional benefits in the form of reducing the consumption of materials for production and reducing the amount of waste generated. There are many physical and chemical procedures useful for the synthesis of NPs and biocompatible hybrid materials with different morphology (shape, size) and properties. Currently, however, methods are being sought to obtain NPs useful for specific applications, e.g., in medicine or catalysis. Examples include specially synthesized nanomaterials used in medicine for personalized anti-cancer therapies; drug-delivery systems, such as quantum dots (QD); and early diagnosis [181]. The enthusiasm for the use of NMs diminishes due to reports of their toxicity, including neurotoxicity; immunotoxicity; hepatotoxicity; reproductive toxicity; and nanoparticle-induced damage to the lungs, kidneys, and other organs [182,183,184]. Nanotoxicology is a new field that only began to develop intensively after 2012, although the first reports of nanotoxicity date back to the beginning of the 21st century [185].

There is no doubt that people today are exposed to nanostructures. Consumer products based on nanotechnology are common. They are found in food packaging, fabrics, home appliances, electronics, medicines, cosmetics, building materials, etc. The anthropogenic environment is polluted as a result of industrial production, which releases more than 300,000 tons of NMs per year, in addition to the use of NMs for protection and treatment, e.g., the remediation of contaminated soil and water [186]. Thus, there are different routes of exposure to nanostructures, i.e., intravenous, dermal, subcutaneous, inhalation, intraperitoneal, and oral [187]. Inhaled NPs travel to various organs and are detected in the lungs, liver, heart, spleen, and brain [188]. It has been shown that the mean half-life of NPs in the lungs is approx. 700 days [189]. Following intravenous injection, NPs have been detected in the colon, lung, bone marrow, liver, spleen, and lymphatic system [188]. When injected intraperitoneally, they pass through the placenta or through the peritoneal cavity into the uterus [190]. After oral administration, they are partially excreted in the feces, and partially, after crossing the gastrointestinal barrier, they enter the general circulation and accumulate in the kidneys, liver, spleen, lungs, and brain [188]. The excretion of NPs takes place via macrophage-mediated phagocytosis. The removal of NPs from the systemic circulation takes place in the liver and spleen [191], and from the lungs—after opsonization—in the alveolar area [191,192].

Due to the increased risk of human exposure to NPs, recent studies have focused on the safety issue of manufactured NMs, i.e., nanotoxicology, which studies the interactions of NPs with biological systems [193,194]. There are reports in the literature that describe how NPs are translocated and how they can destabilize various physiological systems. Researchers suggest that exposure to NPs may stimulate the secretion of cytokines or enzymes that affect the entire body and are not limited to the implant site or a single organ [195,196,197].

Reports on the toxicity of NPs used in medicine are particularly worrying [198]. An example is nanosilver (nano-Ag), which is used in the production of wound dressings and surgical sutures to protect against infection. Unfortunately, studies have confirmed the cytotoxicity of these materials against keratinocytes [199,200,201,202,203,204,205] and mammalian germline stem cells [206]. Therefore, exposure to nano-Ag in cosmetic or disinfecting products may cause fertility problems.

Similar results have been obtained in other in vitro studies in rat liver cell BRL 3A [180], a neuroendocrine cell line (PC-12 cells) [207]. It seems that the cytotoxicity of nano-Ag is related to the deterioration of mitochondrial function by generating oxidative stress (increase in ROS). Interactions of Ag-NPs with the thiol groups of glutathione, thioredoxin, SOD, and thioredoxin peroxidase are probably responsible for the deactivation of the antioxidant defense of the cell at the molecular level [208].

Toxicity has been found to be strongly correlated with particle size, surface area, and aggregation processes, possibly due to a change in surface properties [209]. In addition, smaller NPs (1–50 nm) penetrate biological barriers more easily and bioaccumulate compared to those of larger sizes (51–300 nm) [210]. Chen et al. [211] demonstrated the toxicity of nano-Cu, and other researchers have demonstrated the appearance of pulmonary toxicity by nano-TiO_2_ [212,213,214,215,216] or nano-vanadium oxide (nano-V_2_O_3_) [217], which are harmless at the microscale.

The fate of NPs in the body, i.e., absorption, distribution, metabolism, and elimination from the body (ADME), is determined, apart from the size or shape, by the presence of functional groups on their surface [218,219]. An important role in nanotoxicity is played by protein-NP interaction, which is formed as a result of contact with the body. Although the composition of “the protein crown” depends on the type of NPs, it turns out that most NPs have the ability to bind with apolipoproteins [220]. The resulting complex is able to easily penetrate into the cell and even move to the brain, e.g., with the participation of apolipoprotein E [221]. The final reproductive toxicity is the result of the physico-chemical characteristics of NPs, as well as the exposure time and the route of exposure.

The effects of nanotoxicity have been included in review articles [222,223,224]. A Web of Science database bibliometric analysis of reproductive toxicity reported in 2006–2016 was prepared by Wang et al. [184]. Valuable data linking reproductive disorders to exposure to NMs was also included in a report prepared by the European Chemicals Agency in 2020 [182]. The report collected 111 publications covering 19 NMs. However, most of the studies performed were on specific NPs, and toxicity studies were conducted with mammalian models (in vivo and in vitro assay methods) such as mice and rats, which exhibit genetic similarities to humans. Such data are contained in a review by Brohi et al. [222]; a review by Makato Ema et al. in 2017 [225] on nano-Ag, and earlier in 2010 on the toxicity of manufactured NMs [204]; and a review by Dantas et al. [226] only covering rodent models.

### 3.1. Nanotoxicity and Male Fertility

The barriers in the reproductive system, such as the blood–testes barrier (BTB), play a key role in protecting the testicles from all toxic xenobiotics. Meanwhile, it turns out that NPs reach the reproductive system by exceeding BTB, causing anomalies in the reproductive system. Upon exposure, NPs may accumulate in the testes and epididymides, disrupting the spermatogenesis process [227,228]. Spermatogenesis is a process involving germ cell proliferation and differentiation, leading to the production and release of spermatozoa from the testes. The hormonal and nutritive components necessary for the development and viability of germ cells are provided by Sertoli cells. Sertoli cells not only promote spermatogenesis inside the seminiferous tubules but are also a protective barrier preventing toxins from entering the germ cells [229]. The accumulation of NPs in the testes of males was found after exposure to NPs by various routes, by ingestion, through the skin, inhalation, and injection. Falchi et al. in 2018 [230] drew attention to the influence of NPs on male fertility. While the negative effects of Au-NPs, Ag-NPs, TiO_2_-NPs, etc., on steroidogenesis, spermatogenesis, and fertility in laboratory animals are known, data on male fertility of reproductive age are limited. In order to explain the harmful effects of NPs on reproduction, reproductive indicators and sperm parameters are examined: sperm count, motility and morphology, histopathology of the seminiferous tubules, and testosterone levels.

A bibliometric report prepared in 2021 showed that China is the undisputed leader in research on male reproductive nanotoxicity [185]. In vivo studies are mainly performed with rats (50%) and mice (48%) for an acute duration (1–14 weeks), depending on the test species. NPs are administrated orally (42%) and, less commonly, intraperitoneally and intravenously. In vitro models use testicular cells, mostly sperm cells (38%), Sertoli cells (24%), and Leydig cells (15%), with a 24 h exposure period. The vast majority of studies conducted until 2021 concern the nanotoxicity of Ag.

**Figure 2:**
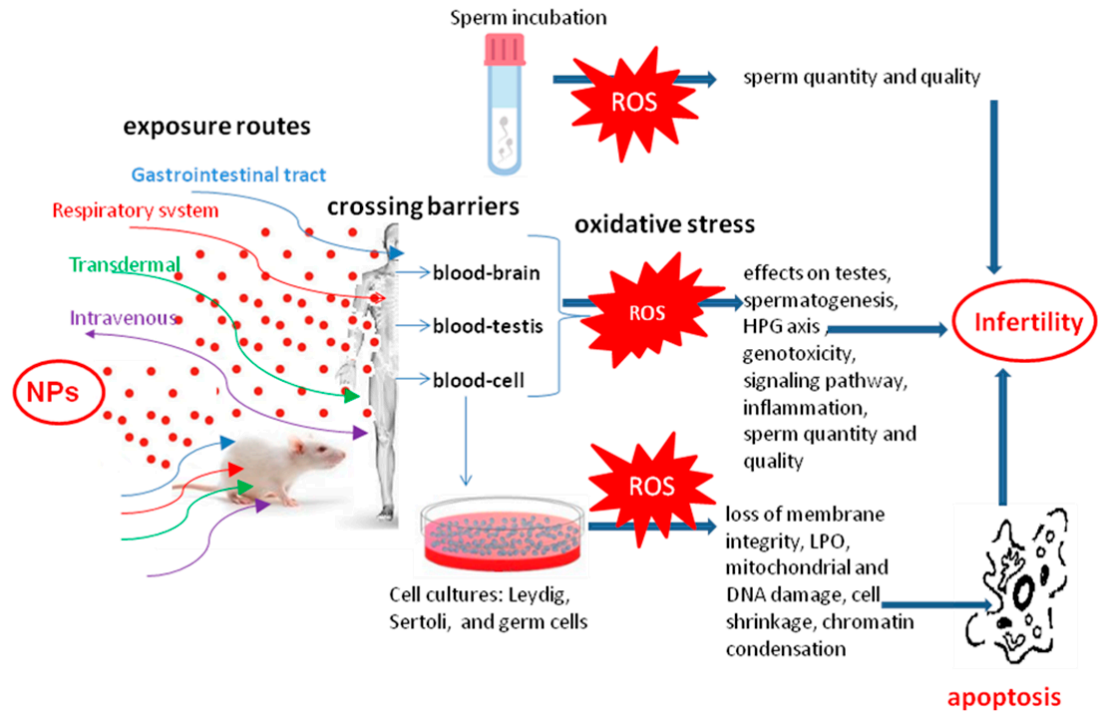
The effect of NPs on the fertility of in vivo and in vitro male models.

The schematic representations of studies on the adverse effects of NPs on the male reproduction system in vivo and in vitro are summarized in Figure 2 and Table 3 [228,229,230].

#### 3.1.1. The Sperm

The influence of NPs on sperm quality has only been studied since the beginning of the 21st century. The in vitro toxicity of NPs was achieved by the direct addition of NPs to semen. The physical characteristics of the semen were investigated, and they played a key role in the transfer of genetic material [231]. Sperm DNA integrity and cell membrane integrity were compared before and after NPs exposure. The research results obtained by various authors are inconsistent and depend primarily on the type of nanoparticles used. It should be emphasized that there is little research completed on human material.

The first study on magnetite NPs was carried out by Ben-David Makhluf et al. [232]. In this study, the transfer of Fe_3_O_4_-NPs coated with polyvinyl alcohol (PVA) into cattle sperm, namely, the mitochondria in the tail and acrosomes in the head, was confirmed. However, the authors did not find an effect of the Fe_3_O_4_-PVA colloidal solution on the mobility or the ability to fertilize the egg, i.e., the efficiency of the acrosome reaction.

Three years later, in 2009, Wiwanitkit et al. [233] proved the spermatotoxicity of Au-NPs while suggesting that other NPs should also be thoroughly tested for spermatotoxicity. As a result of mixing a suspension of Au-NPs (9 nm) at a concentration of 44 μg mL^−1^ with male semen, a loss of motility in 25% of the sperm was observed. The authors also described the fragmentation of sperm and the penetration of Au-NPs into the sperm head and tails. This study explained the reported infertility and epididymitis in men exposed to Au-NPs [234]. The high toxicity of nano-Au may be due to an increase in activity when NPs are deposited on matrices such as metal oxides or activated carbon. This phenomenon is used in the case of chemical reactions, such as CO oxidation and propylene epoxidation [235].

In vitro studies on the effect of nanotoxicity on male reproduction are performed on spermatogonial stem cell lines isolated from mouse and cattle sperm. One of the reports concerns diesel exhaust particles (DEP), soot (CB), and TiO_2_-NPs, which were tested on mouse Leydig TM3 cell lines, or testosterone-producing testosterone cells [236]. The research was performed by an advanced analytical technique using Transmission Electron Microscopy (TEM) and Scanning Electron Microscopy/Energy-Dispersive X-ray Spectroscopy (FE-SEM/EDS). The TiO_2_-NPs turned out to be the most cytotoxic. It should be emphasized, however, that all tested nanoparticles were captured by Leydig cells and influenced the viability, proliferation, and expression of genes, i.e., heme oxygenase-1 (HO-1), which is a marker of oxidative stress, and the gene for steroidogenic acute regulator protein (StAR), that is, the factor controlling the mitochondrial transfer of cholesterol. Another study was performed by Braydich-Stolle et al., in 2005 [206], on the C18-4 cell line, which was previously established from type A spermatogonia isolated from 6-day-old mouse testes, examining the toxicity of Ag-NPs (15 nm), MoO_3_-NPs (30 nm), and Al-NPs (30 nm). The results clearly showed the influence of NPs concentration on toxicity. The authors proved that Ag-NPs were the most toxic, while MoO_3_-NPs were the least harmful. To rank NPs for toxicity with respect to mitochondrial function and plasma membrane integrity MTS [[3-(4,5-dimethylthiazol-2-yl)-5-(3-carboxymethoxyphenyl)-2-(4-sulfophenyl))-2H-tetrazolium] reduction assay, a lactase dehydrogenase (LDH) leakage assay and the activation of apoptotic pathways were used. It turned out that Ag-NPs decreased mitochondrial function and cell viability. The EC_50_ value calculated for Ag-NPs was the lowest and was 8.75 µg mL^−1^, while the EC_50_ value for MoO_3_-NPs was 90 µg mL^−1^. The authors emphasized the fact that Ag-NPs interfere with cellular metabolism and may promote cell apoptosis. A significant increase in LDH leakage was observed for Al-NPs, for which the EC_50_ was 4.7 μg mL^−1^, and for MoO_3_-NPs, with the EC_50_ value of 5 μg mL^−1^.

Another study assessing the effect of NPs on the proliferation of mouse seminal stem cells (SSCs), which are the source of adult testes germline, is the work of Braydich-Stolle et al. in 2010 [237]. At concentrations above 10 µg mL^−1^, Ag-NP induced a significant decrease in SSC proliferation through specific interaction downstream of the Ret Fyn kinase. Undoubtedly, most NPs have harmful or toxic effects on spermatogenesis. Exposure to Au-NPs [233], silica [50], TiO_2_-NPs [238], and many other manufactured NMs have been shown to be toxic to sperm.

However, some NPs show a beneficial or nontoxic effect on spermatogenesis. Several reports prove the beneficial effect of NPs on sperm parameters (number, motility, viability, and percentage of live sperm). Kobyliak et al. [239] reported that cerium dioxide (CeO_2_) NPs lowered the level of oxidative stress in rat sperm, as evidenced by improved sperm parameters and decreased serum lipid peroxidation product levels, as well as increased CAT and SOD activity. The above observation was confirmed by a study [240] that described the beneficial effect of CeO_2_-NPs on kinetic and morphological parameters of ram semen, such as membrane mobility and integrity, as well as the absence of genotoxicity. CeO_2_-NPs seem to have a future in medicine due to their ability to store oxygen and the activity of scavengers against ROS comparable to antioxidant enzymes in biological systems [241]. Numerous reports describe a reduction in ROS levels in tissues or cells after exposure to CeO_2_-NPs [242,243]. In contrast, some authors have observed a pro-oxidative [244] or DNA-damaging effect in liver cells and leukocytes [245]. It is likely that CeO_2_-NPs may show different activity in the reproductive system depending on the physico-chemical properties, concentration, or duration of exposure, which would explain the observed discrepancies. This does not change the fact that CeO_2_-NPs are still controversial. In mice, exposure to CeO_2_-NPs led to a reduction in fertilization and accumulation in granule cells and sperm plasma membranes [246]. In turn, sheep gametes tolerated well the coincubation with CeO_2_-NPs. Granular cells are likely to internalize this compound by endocytosis, possibly in this case [247].

Another example is the use of Zn-NPs as an antioxidant in Holstein bulls’ semen extender [248]. The authors of the study assumed that because Zn is important for testicular development and spermatogenesis, it can be expected that Zn-NPs will not only be toxic but may be effective in preserving sperm quality during storage. The study indeed demonstrated the effectiveness of Zn-NPs in defending sperm from lipid peroxidation, which results in DNA damage, decreased sperm parameters, gene expression, and defective membrane integrity. It has been reported that also nano-Se diet supplementation produced positive effects on sperm quality in male goats [249,250]. Similarly, DMSA-coated maghemite NPs [251] showed no adverse effects on germ cell kinetics, sperm structure, and function.

#### 3.1.2. NPs Accumulation in Testes

Accumulation of NPs in the testes of males was found after exposure to NPs by various routes: oral [252], intragastric [253], intraperitoneal, or intravenous [254]. However, the intramuscular administration of silica–gold NPs [255] or TiO_2_-NPs for 4 weeks at concentrations ranging from 0.1 to 10 mg/kg BW did not result in an accumulation of NPs in the testes of mice [256].

The main factors influencing toxicity are the lipophilicity of the xenobiotic and the related possibility of penetration through tissue barriers. NPs can penetrate tissue barriers and accumulate in the testes, as confirmed in animal models, but are not always accompanied by a visible toxic effect, as in the case of silica-coated magnetic NPs administered at a dose of 10, 25, 50, or 100 mg/kg to male mice [257] or silica–gold NPs by intramuscular injection into mice [258].

The most common, however, is the occurrence of toxic effects following the accumulation of NPs in the testes and the resulting male infertility. Examples include exposure to Ag-NPs [259] or to TiO_2_-NPs [260], which leads to altered testicular histology and reproductive toxicity in animal models. Modification of the surface of nanoparticles, e.g., ω-Methoxy and ω-aminoethyl poly (ethylene glycol)-modified (PEG-NH2 @ AuNP), showed that NPs could accumulate in mouse testes and enter germ cells; however, there was an increase in plasma T levels and no effect on male fertility, fetal survival, or fetal development [261].

#### 3.1.3. Spermatogenesis

Spermatogenesis begins in the testicles’ seminiferous tubules. Exposure to NPs has been shown to influence this process, contributing to a reduction in sperm count [253,262]. The influence of NPs takes place at the molecular level and concerns the change in the overall expression of genes involved in spermatogenesis [263]. Such studies were conducted by Hong et al. [264], who confirmed alterations of testes-specific gene expression in male mice following nano-TiO_2_ exposure. In their further studies [260,265], they confirmed decreases in spermatogenesis via biochemical dysfunctions in the testes and the apoptosis of primary cultured Sertoli cells exposed to TiO_2_-NPs.

NPs such as TiO_2_-NPs are dangerous to male offspring in utero [266]. It turns out that the subcutaneous injection of 400 μg TiO_2_-NPs in pregnant mice harms the development of the male reproductive system. Fetal depletion of Sertoli cells, the rupture of the seminiferous tubules, altered testicular morphology, and decreased daily sperm production and epididymal motility were observed in the fetus. TiO_2_-NPs could be found in Leydig cells, Sertoli cells, and sperm in the testes of male offspring as early as 6 weeks postpartum.

In a study by Yuan et al. [267], the expression levels of 44 imprinted genes were analyzed by quantitative real-time PCR in TM-4 Sertoli cells after a low dose of (10 nM) Au-nanorods treatment for 24 h. The authors reported, among others, diminished expression of Kcnq1, Ntm, Peg10, Slc22a2, Pwcr1, Gtl2, Nap1l5, Peg3, and Slc22a2, while Plagl1 was significantly overexpressed. As demonstrated by the example of Ag-NPs [268], the internalization of NPs into spermatozoa may alter sperm physiology, leading to poor fertilization and embryonic development. More recently, perspectives of NPs in male infertility have been described in some review papers [223,269,270,271].

In contrast to most reports presenting reproduction nanotoxicty, a study by Eman T. Hamam et al. in 2022 [272] described the use of ZnO-NPs with the aim of restoring male rats’ reproductive capacity after the induction of Cisplatin (Cis) by promoting spermatogenesis. The study raised hopes for tackling the impaired initiation of spermatogenesis by Cis used in cancer treatment. ZnO-NPs are used in many products and are considered safe despite inconsistent reports of toxicity and unfavorable health effects. A meta-analysis performed in 2022, based on a systematic review of 76 publications, revealed several attributes responsible for cytotoxicity after exposure to ZnO-NPs. Based on the results of in vitro studies, it was established that nanotoxicity strictly depends on the dose and size of NPs, exposure time, and the type of cell line [273]. Examples of NPs and their effects on reproductive function in males are collected in Table 4.

## 4. Conclusions and Future Perspectives

Nowadays, the reproductive health of the human species is at considerable risk. Most cases of male infertility are caused by poor sperm quality of unknown etiology. One of the causes of suboptimal sperm quality is oxidative stress, which damages sperm DNA. In the late twentieth and early twenty-first centuries, research on infertility focused on the role of environmental factors. Many reports demonstrated the toxicity of heavy metals and described the mechanisms of their action on the reproductive system. Potentially harmless factors were given little attention. The current review collected data on the effects of metallic NPs that dominate in modern industry and essential metals, which in excess also cause changes in organ weight, abnormalities in sperm and seminiferous tubules, and abnormalities in spermatogenesis and hormones/gens/enzyme involved in sperm production. Several observations emerged from the collected material that may inspire future research:

(i) Despite the existence of extensive data on exposure to single metals or metallic NPs, there is a gap in the safety assessment of multimetals, both on the pituitary–nucleus axis and sperm. There is a scarcity of such multi-element studies. An example is [296], which studied male Wistar rats’ exposure to Zn, Al, and Cu. To our knowledge, there are no studies that have assessed the effects of Multi NPs on the reproductive system.
(ii) Most in vivo studies use the oral route of administration via the gastrointestinal system, while other routes of exposure are less frequently used. An example is a transdermal exposition, which is important in studying the absorption of NPs from cosmetics that are absorbed slowly through the skin into the body.
(iii) NPs are able to cross biological membrane barriers, including the blood–testis barrier. The harmfulness of most NPs to male fertility (spermatogenesis) suggests extreme caution regarding the use of NPs in medicine (e.g., Nano-Ag).
(iv) The cell membrane, as a negatively charged phospholipid bilayer, is generally not an obstacle for cationic nanoparticles. The functionalization of nanoparticles with appropriate ligands may improve or hinder their entry into cells.
(v) The nanotoxicity of NPs depends on properties (i.e., composition, size, shape, and functionalization). The available reports rarely track the reproductive toxicity of functionalized NPs [214,215]. According to recent reports, the NPs’ surface, due to high reactivity, adsorbs proteins [297], plant metabolites [298], etc. Therefore, differential responses related to NPs surface properties can be expected.
(vi) Since most studies are performed in vitro or in vivo in laboratory animals, no data are available on the effects of long-term exposure. Similarly, there is a lack of distinction between the adverse effects of the trace elements and nanomaterials on male fertility as temporary or permanent.
(vii) Although significant advances have been made in the treatment of male infertility, e.g., by using multi-element supplementation, there is still no public awareness of the effects of an overdose of essential metals such as Se, Fe, or Zn on reproductive hormones and sperm quality.

## Figures and Tables

**Table 1:** Concentrations of trace elements (mg L^−1^) in normal semen specimens or other biological materials taken from fertile men.

Specimen	n	Ca	Mg	Zn	Se	Fe	Cu	Other Metals	Ref.
Blood	96	48.7–52.0	73.7–76.6	6.76–7.10	0.230–0.246	449–469	0.89–1.00	Pb, Cd, Hg	[78]
Serum		33.5–36.0	22.0–24.2	1.21–1.38	0.064–0.068	3.00–3.40	0.62–0.68		
SP		59.0–62.3	71.7–75.4	138–152	0.034–0.035	269–283	148–165		
SP	482	-	-	0.117	0.026	0.121	0.063	18 metals	[79]
SP	19	240.49 ± 50.5	60.85 ± 10.89	140.08 ± 20.01	-	-	-	Na, K	[80]
SP	47	-	-	1.24–1.53	-	-	-	-	[81]
Semen	22	-	-	-	0.054 ± 0.023	-	-	Cd, Pb	[82]
SP		-	-	-	0.040 ± 0.016	-	-		
SP	40	-	13.14 ± 3.65	141.7 ± 30.23	0.061 ± 0.018	-	165.56 ± 40.13	-	[83]
SP	-	-	-	18–301	0.021–0.191	0.05–0.63	0.03–0.3	Mn, I, Pb, Cd, Mo	[84]
Semen	97	-	-	7.626 ± 0.090	-	-	-	-	[85]
Serum ^1^	30	93.09	22.07	3.53	0.49	2.26	0.90	Na, K	[86]
SP ^1^		103.78	25.72	1.10	0.09	2.66	0.87		
SP	28	-	-	-	0.07 ± 0.020	-	0.195 ± 0.045	Mn, Pb, As	[87]
SP	96	-	550.12 ± 282.51	188.42 ± 99.61	-	2.02 ± 0.74	1.29 ± 0.58	-	[88]
SP	64	-	-	0.127 ± 0.075	0.012 ± 0.019	-	0.041 ± 0.041	As, Sb, Hg, Al, Cd, Ni, Pb, V Mn, Ti, Cr, Mo,	[89]
SpermDNA		-	-	0.018 ± 0.042	-	-	0.00011 ± 0.0003	-	
Blood	30	-	-	0.213 ± 0.139	-	-	0.107 ± 0.084	Pb, Cd	[90]
SP		-	-	0.131 ± 0.107	-	-	0.106 ± 0.094		

^1^ Data obtained for bulls. SP—seminal plasma.

**Table 2:** The role of Zn in male fertility and the effects of deficiency [76,77,80,92,93,94,95,96,97,98,99,100,101,102,103,104,105,106,107,108,109,110,111].

Organ or System/Role	Action	Zn Deficiency
HPG axis/ hormone production	Inhibition of 5α reductase and affinity to LH receptor	Low serum T, testicular failure, changed sex steroid hormone receptor levels, damaged LH receptors, increase in circulating LH, decrease in T synthesis in Leydig cells
Antioxidant defense system/ free-radical scavenging	Inhibition of DNases and activity of Cu/Zn SOD	Oxidative damages (lipids, proteins, DNA), increase in LPO, increased MDA in the serum and seminal plasma and reduced levels of SOD, damage to the Leydig cells, apoptosis
cell physiology/ anti-apoptotic agent	Inhibition of caspases, Bcl-2/Bax ratio increase	DNA fragmentation, apoptosis, decreased population of the Leydig cells, germ cells, cell and tissue death
Epigenetics/ gene regulation, DNA methylation	Zn expression Zn transport binding proteins, testis-GC specific genes	Reduced reproductive potential, delayed sperm maturation
Testes/ testes development	participation in spermatogenesis (mitosis of spermatogonia and spermatocyte meiosis)	Retarded genital development, reduced testes weight, changes in the structure of Leydig cells, lower sperm concentration of the ejaculate, hyperviscosity of semen
Spermatozoa physiology/ cell metabolism	lipid and protein metabolism, oxygen consumption, nucleic acid synthesis, epithelial membrane integrity, chromatin condensation	Abnormal morphology, count, viability, motility of sperm, head–tail attachment problem, inhibition of spermatid differentiation, dysfunction of the zinc finger motif Cys2/His2 of P2 protamines
Fertilization/ embryonic formation	capacitation, the acrosome reaction	Change in pH, proteasomal activities, transfer of the amino peptidase from prostasomes, lower sperm membrane fluidity, improper fertilization

Abbreviations: deoxyribonucleases (DNases).

**Table 3:** Influence of NPs on the male reproduction system. Based on Refs. [228,229,230].

Model	Exposure	Cell Lines	Accumulation	Observations
In vitro Sperm (boar, buffalo, bull, human, mouse, ram, pig)	0.05–1000 µg/mL/very short (<24 h), short (24–72 h), and long time (>96 h).	Germ cells: LCs, TM-3, PTCs, SCs, ISP-1, SSCs, SCs-GCs, CPCs, SPTs, C18-4	Plasma membrane, head, midpiece, the tail of sperm, cytoplasm, cytoskeleton, nucleus, mitochondria	Damage to membrane integrity, potential, ion permeability, signal transduction; abnormal morphology (absent acrosome, head, disrupted chromatin heads, curved midpieces, tails), LPO, mitochondria (reduction in oxygen consumption and ATP levels); damage to mtDNA, the polymorphic profile of DNA; morphological changes (cell shrinkage, chromatin condensation, disorganization of microtubule network, necrosis); down-regulation of genes involved in cell proliferation, meiosis, and differentiation; induction of autophagy and apoptosis; reduced motility in spermatozoa, defects in acrosomal reaction, the ability to penetrate the oocyte, and blastocyst formation
		Sertoli cells	Cytoplasm, nucleus	Loss of membrane integrity, membrane potential (dysfunction of BTB-related proteins), morphological changes (cellular and nuclear shrinkage, chromatin fragmentation, endoplasmic reticulum expansion, mitochondrial swelling, accumulation of autophagosomes, disordered microfilament networks), mitochondrial damage, DNA damage, decrease in cell viability, cell apoptosis
		Leydig cells	Cytoplasm, nucleus	Steroidogenesis inhibition (testosterone production), morphological abnormalities, LPO, loss of membrane integrity, mitochondrial damage, DNA damage, reduced viability, autophagy, and apoptosis
In vivo White rabbit, rat (albino, Fisher, Sprague Dawley), Wistar	(0.001 to 2000 mg/kg)/acute (1–14 days), subacute (repeated doses 14–28 days), subchronic (repeated doses 28–90 days), chronic (repeated doses 90 days)	Testes	Brain, organs of the reproductive system (testes, epididymis, prostate, seminal vesicle, the seminiferous tubules, interstitial Leydig cells, Sertoli cells, spermatogonia, spermatocytes, spermatids, sperm cells), other organs (liver, spleen, kidneys, lungs)	Fertility rate decrease; testicular weight reduction; sperm quantity/quality (increase in sperm DNA damage, morphological abnormalities, motility, mitochondrial activity, and acrosome integrity); histopathological changes (intracellular vacuolations; degeneration, atrophy, and necrosis of germ cells, Sertoli cells, and Leydig cells; irregularities in plasma membrane; nuclear chromatin loss; mitochondria swelling and cristae disappearance; dilated endoplasmic reticulum; and increase in lysosomes), morphometric alterations of germinal epithelium; decline in the number of spermatogonia, spermatids, Sertoli and Leydig cells; impairments in spermatogenesis (reductions in germ cell content, reductions in daily sperm production and sperm count in testes and epididymis); changes in the expression of apoptosis-related proteins; changes in testosterone levels (plasma/serum, intratesticular testosterone)
		HPG axis		Hormonal imbalance (GnRH, LH, FSH, prolactin, inhibin, DHT, estrogen, testosterone); changes of the transcript expression of genes involved in the regulation of the HPT axis (Gnrh, Esr1, Esr2, Ar, Inhbb) in hypothalamic, pituitary, and testicular tissues
		epididymis		Histopathological changes (hyperplasia of epithelial cells, the lining of the duct of epididymis, cell pyknosis, necrosis and abscission, vacuolar cytoplasm in the cauda of principal cells, increased fibrotic tissues, infiltration of connective tissues and inflammatory cells, and interstitial congestion with ducts presenting empty lumen lacking spermatozoa) Decrease in the mitochondrial activity of epididymal sperm (reduction in the number of sperm in the lumen of epididymal duct, change in epididymis weight) and changes in the epididymal sperm (reduced sperm concentration in the cauda, motility, and acrosome integrity, and increases in DNA damage)

Abbreviations: gonadotropin-releasing hormone (GnRH), luteinizing hormone (LH), follicle-stimulating hormone (FSH), dihydrotestosterone (DHT); hypothalamus–pituitary–gonad axis (HPG axis); lipid peroxidation (LPO); mitochondrial DNA (mtDNA); blood–testes barrier (BTB).

**Table 4:** The impacts of selected NPs on reproductive function in males.

Model	Expose	Doses	Findings	Ref.
**Copper oxide nanoparticles (CuO-NPs)**				
Male albino mice BALB/c	Oral intake	25, 35 mg/kg BW	Reduced proliferative activity and differentiation in the potential of epithelial cells; reduction in the number of Leydig cells, the incidence of necrosis, damage in organs (testes, epididymis, and seminal vesicles); spermatogenesis: low number of sperm, distorted sperm leading to the formation of embryos with some abnormalities; MDA and caspase-3 increased; Ki67 protein decreased; CD68 protein increased; reduction in the seminal vesicle, increased prostate size	[274]
**Zinc oxide nanoparticles (ZnO-NPs)**				
New Zealand rabbit spermatozoa	Incubation	6–391 mg/mL per 0, 1, 2, 3 h	Spermatozoa membrane integrity decrease, negative dose-dependent effect on spermatozoa motility and viability	[275]
Male mice	Oral administration	50, 150, 450 mg/kg for 14 days	Detachment, atrophy, and vacuolization of germ cells; vacuolization of Sertoli and Leydig cells; decrease in the number of sperm in the epididymis; decrease in the concentration of T in serum; up-regulated IRE1α, XBP1s, BIP, and CHOP genes; increase in the expression of caspase-3; reduced body weight; increased relative testicular weight and relative epididymis weight in a dose-dependent manner	[276]
Adult albino rats	Oral subacute	422 mg/kg/day for 4 weeks	Congestion in blood vessels; detached germinal epithelium from the basement membrane; absence of spermatozoa in seminiferous tubules; fragmentation of DNA in testicular and prostatic tissue; increase in the mean area percentage of iNOs immunoreactions; testicular and prostatic tissue inflammatory cytokines; elevation in serum level of MDA; reduction in GSH, CAT, and SOD activities	[277]
Albino rats	Orally	100 and 400 mg/kg/day for 12 weeks	Disorganization, vacuolation, and detachment of germ cells in testicular tissue; decrease in sperm cell count, sperm motility, live percentage of sperm and normal sperm; decrease in serum testosterone level and antioxidant enzymes activity (SOD and CAT) and the GSH-Px level; increase in LPO in the affected testes; decrease in 3β-HSD, 17β-HSD, and Nr5A1 transcripts; quercetin—beneficial for preventing or ameliorating ZnO-NP reproductive toxicities	[227]
Male NMRI mice	Orally	5, 50, 300 mg/kg/day for 35 days	Spermatogenetic factors: change in the number and motility of sperm, decrease in the diameter and height of the seminiferous tubules, blocking of the maturity of sperm cell lines, epithelial vacuolization, increase in sloughing of germ cells and their detachment, formation of multinucleated giant cells in germinal epithelium	[278]
Semen from healthy persons	Incubation	10–1000 µg/mL (37 °C) for 45–180 min.	Dose- and time-dependent cytotoxicity; maximum cell death percentage—20.8%, 21.2%, and 33.2% after 45, 90, and 180 min, respectively Highest concentration (1000 µg/mL) resulted in highest toxicity	[279]
Sertoli (TM-4) and spermatocyte (GC2-spd) cell lines (in vitro models)	-	8 μg/mL (sublethal dose)	Induced oxidative stress in both cells lines (decreased glutathione level and increased MDA level) Down-regulated expression of BTB proteins in Sertoli cells, increased TNF-α secretion, DNA damage in germ cells and GC2-spd cells, S phase arrest, lower expressions of BTB proteins (ZO-1, occludin, claudin-5, and connexin-43)	[280]
Male rats	Intraperitoneal i.p. injected	5 mg/kg once a week for 8 weeks	Group treated with Cis and ZnO-NPs: decrease in ROS BTB proteins restoration, enhanced architecture of the testes, and increased sperm DNA integrity	[272]
**Nickel nanoparticles (Ni-NPs)**				
Male Sprague Dawley rats	Orally	5, 15, 45 mg/kg/day for 10 weeks	Increased FSH and LH, lowered estradiol (E2), serum levels decreased, the ratio of epididymis weight over body weight increased, motility of the sperm changed, diminished shedding of epithelial cells of the raw seminiferous tubules, disordered arrangement of cells in the tubes, cell apoptosis	[281]
BALB/c mice (in vivo),GC-1 cells(in vitro)	Orally/incubation	5, 15, and 45 mg/kg/day	In vivo: serum T, FSH, LH, and sperm count decreased; sperm abnormality; expression of Drp1, Pink1, and Parkin proteins increased; seminiferous tubules of the testes changed In vitro: MMP, ATP, and cell viability decreased; apoptosis; accumulation of ROS; expression of Drp1, Pink1, Parkin, Bax, caspase-9, and caspase-3 proteins; expression of Bcl-2; Bax/Bcl-2 ratio increased	[282]
BALB/c mice	Intratracheal instillation	0.1 mL/10 g once a week for 28 days	Changes in sperm deformity and serum reproductive hormones, apoptotic cells number increased; testicular spermatogenic cells damage; expressions of proteins (Drp1, Pink1, Parkin) increased	[283]
**Silver nanoparticles (Ag-NPs)**				
Wistar rats	Oral exposure	15 and 50 µg/kg BW	Weight not changed, growth less diminished, sperm reserves in the epididymis and diminished sperm transit time, a reduction in sperm production, impairment in spermatogenesis, and lower sperm count	[284]
Wistar rats	Intravenous administration	5 and 10 mg/kg BW	Decrease in epididymal sperm count; increased level of DNA damage in germ cells; change in the testes’ seminiferous tubules morphometry, adipose tissue distribution, and the frequency of abnormal spermatozoa; no alteration in body and organ weight, 20 nm AgNP—more toxic than 200 nm ones	[285]
Male rats	Sub-dermal	50 mg/kg BW for 28 or 7 days	Reduction in BW, decrease in the relative weight of the testes and epididymis with the same dose exposure for 7 days, Ag-NP triggered hormonal imbalance and induced oxidative stress in the testes and epididymis, negatively affecting sperm parameters	[286]
Wistar albino rats	Intraperitoneal injection	100 mg/kg/day for 7 days 1000 mg/kg/day for 7 or 28 days	Congestion of blood vessels, detachment of the germinal epithelium and distortion in seminiferous tubules, reduction in the germinal epithelium, absence of spermatozoa in shrunk seminiferous tubules, tissue damage increased with increased dose and duration of exposure	[287]
Male rats	Intraperitoneally injected	2 or 4 mg/kg BW	Damaging changes of the seminiferous tubules, vacuolation in the seminiferous tubules with a reduced number of spermatogenic cell lines (at a low NPs dose), increased reduction in spermatogenic cell lines with vacuolation in germinal epithelial cells and basement membrane damage, detachment from the surrounding tubules, severe congestion in blood vessels, and few Leydig cells in the interstitial tissue (at high NPs doses), protective role of camel milk for the testes damaged by AgNPs	[288]
Male mice	Intraperitoneally injection	0.2 mL once a week at a dose of 40 mg/kg of BW	Sexual behavior, oxidative defense parameters, sperm count, and motility of the sperm, the apoptotic cells in testicular cross-sections, and TBARS level increased; YO-NPs with protective effects, lowering of Ag-NPs toxicity, no difference in RW	[289]
Spermatozoa (BDF1) mice	Addition	0.1, 1, 10, or 50 μg/mL incubated at 37 °C for 3 h	Sperm viability and the acrosome reaction inhibition in a dose-dependent manner, increased sperm mitochondrial copy numbers, morphological abnormalities, mortality decreased, decrease in the rate of oocyte fertilization and blastocyst formation, lower expression of trophectoderm-associated and pluripotent marker genes in blastocysts	[268]
**Iron oxide nanoparticles (Fe_2_O_3_-NPs)**				
Male mice	Intraperitoneal exposure	25 and 50 mg/kg once a week for 4 weeks	Sloughing and detachment of germ cells and vacuolization in seminiferous tubules of the testicular tissues; accumulation of NPs; increased ROS species, LPO, protein carbonyl content, GPx activity, and NO levels; decrease in SOD, CAT, glutathione, and vitamin C levels; increased serum T levels, expression of Bax, cleaved-caspase-3, and cleaved-PARP; cell apoptosis; damage to the seminiferous tubules; decrease in the number of spermatogonia, primary spermatocytes, spermatids	[290]
**Titanium dioxide nanoparticles (TiO_2_-NPs)**				
Male albino rats	Intraperitoneal injection	300 mg/kg for 14 days	Increases in the thickness of interstitial space, congestion of blood vessels, and detachment of the germinal epithelium from the basement membrane in the seminiferous tubules, beneficial effects of beta carotene administration	[291]
Sertoli cell culture	Incubation	5, 15, or 30 lg/mL for 24 h	Reduction in cell viability, lactate dehydrogenase release, and induction of apoptosis or death of Sertoli cells; elevation in ROS species; reductions in SOD, CAT, and GSH-Px activities; decreases in DWm, release of cytochrome c into the cytosol; upregulation of cytochrome c, Bax, caspase-3, glucose-regulated protein 78, and C/EBP homologous protein and caspase-12 protein expression; and downregulation of bcl-2 protein expression	[260]
**Aluminum oxide nanoparticles (Al_2_O_3_-NPs)**				
Male rats	Oral	70 mg/kg BW	Distortion in seminiferous tubules; wide spaces among interstitial cells (Al_2_O_3_-NPs); irregularity in the seminiferous tubules’ shape, empty lumina; and reduced thickness of the epithelium lining (ZnO-NPs; 100 mg/kg BW); co-administration of Al_2_O_3_-NPs, ZnO-NPs caused severe damage to the seminiferous tubules and basement membrane	[292]
**Gold nanoparticles (Au-NPs)**				
Male mice BABL/c (in vivo), TM3 cells (in vitro)	Intravenously injected	0.17 or 0.50 mg/kg/day 0 for 14 days	Accumulation of NPs in the testes; reduced plasma T; increased rate of epididymal sperm malformation; induced autophagosome formation; enhanced ROS production; disrupted cell cycle, DNA damage, and cytotoxicity in TM3 Leydig cells; inhibition of the synthesis of T in TM3 cells; reduced expression of 17α-hydroxylase	[293]
Male bulb-c mice	Intraperitoneal injection	40 and 200 µg/kg/day for 7 and 35 days	Sperm motility and morphology decrease, increase in abnormal spermatozoa (TB, AB, and CMA3), increase in instability of chromatin and the rate of sperm DNA damage	[294]
TM-4 Sertoli cells	Addition of gold nanorods	0 and 10 nM for 24 h	Decreased glycine synthesis, membrane permeability, mitochondrial membrane potential, and disruption of BTB factors in TM-4 Sertoli cells; aberrant expression of imprinted genes in TM-4 Sertoli cells.	[267]
**Zinc nanoparticles (Zn-NPs)**				
Holstein bull semen (in vitro, in vivo)	Incubation	10^−6^–10^−2^ M	Plasma membrane integrity improved in the semen, proportions of live spermatozoa with active mitochondria increased, level of MDA lowered, Sperm—total and progressive motility, sperm viability increased DNA fragmentation, pregnancy rate—not changed, blastocyst rate increased, embryo development rate (in vitro)—no change	[248]
**Manganese Dioxide nanoparticles (MnO_2_-NPs)**				
Albino Wistar rats	Sub-chronic injection	100 mg/kg/day for 4 weeks	Decrease in the number of sperm, spermatogonia, spermatocytes, the diameter of seminiferous tubules, motility of the sperm; no difference in the weight of prostate, epididymis, left testicle, estradiol, and T levels	[295]
**Selenium nanoparticles (Se-NPs)**				
Taihang black goats	Oral	0.3 mg/kg	Increase in final BW, total blood, serum and tissue Se concentration, serum GSH-Px, SOD, CAT, MDA	[249]
Male Boer goats	Oral	0.3 mg/kg for 12 weeks	Testicular Se level, semen GPx, and ATPase activity increased; semen volume, density, motility, and pH—not affected; membrane system integrity improved; positive effects of nano-Se diet supplementation on sperm abnormality, abnormal spermatozoal mitochondria, membrane system integrity	[250]
**Cerium oxide nanoparticles (CeO_2_ NPs)**				
The ejaculates of Sarda rams	Incubation	0.44 and 220 μg/mL	A beneficial effect on motility parameters, the velocity of sperm cells enhanced, beneficial effects on the integrity of plasma membranes of spermatozoa, no change in production of ROS after 96 h of incubation—at 4 °C, the integrity of DNA was constant	[240]

Abbreviations: body weight (BW); relative weight (RW); glutathione peroxidase (GSH-Px); superoxide dismutase (SOD); catalase (CAT); malondialdehyde (MDA); aniline blue (AB); toluidine blue (TB); chromomycin A3 (CMA3); blood–testes barrier (BTB); cisplatin (Cis); reactive oxygen species (ROS); follicle-stimulating hormone (FSH); luteinizing hormone (LH); testosterone (T); lipid peroxidation (LPO); thiobarbituric acid reactive substances (TBARS); endoplasmic reticulum stress (ER stress); yttrium oxide nanoparticles (YO-NPs); mitochondrial membrane potential (MMP); adenozyno-5′-trifosforan (ATP); mitochondrial membrane potential (DWm).

## Data Availability

Not applicable.
